# Surgical Management of an Atypical Presentation of a Thyroid Storm

**DOI:** 10.5812/ijem.13539

**Published:** 2014-04-01

**Authors:** Ricardo Mario Aulet, Richard O. Wein, Richard D. Siegel

**Affiliations:** 1 Tufts University School of Medicine, Boston, USA; 2 Department of Otolaryngology-HNS, Tufts Medical Center, Boston, USA; 3 Department of Endocrinology and Diabetes, Tufts Medical Center, Boston, USA

**Keywords:** Thyroid Crisis, Thyroidectomy, Graves' Disease, Antithyroid Agents

## Abstract

**Introduction:**

Thyroid storm is a rare complication of Graves' disease that can carry a poor prognosis. In order to prevent major complications, thyroid storm must be quickly identified in patients and treatment must be promptly implemented. Medical treatment is usually initiated with antithyroid medications, such as propylthiouracil (PTU), methimazole, and beta-blockers. However, some patients may experience adverse reactions to these medications and alternate treatment options must be explored.

**Case Presentation:**

We report a case of a 30-year-old female initiated on PTU after diagnosis with Graves' disease that later presented an acute thyroid storm.

**Discussion:**

Therapy was changed to methimazole, yet the patient subsequently developed angioedema and dyspnea. Medical management was discontinued and emergent thyroidectomy was performed without complication.

## 1. Introduction

Hyperthyroidism is a condition that affects an estimated 2.1% of the population (1). Thyroid storm is a rare, life threatening condition characterized by severe clinical manifestations of thyrotoxicosis (2). While its exact incidence in the United States is unknown, a national survey from Japan estimated an incidence of thyroid storm in hospitalized patients to be 0.20 per 100,000 patients, per year (3). Thyrotoxic storm carries a mortality rate of 10-50% and treatment should be initiated immediately after suspicion (4, 5). In order to make an accurate and prompt diagnosis, a set of criteria are used, that include central nervous system effects, gastrointestinal dysfunction, heart rate, signs of congestive heart failure, atrial fibrillation, and any precipitating events (6).

Grave’s disease is the most common cause of hyperthyroidism comprising approximately 60% of all cases (7). Initial treatment for this condition includes antithyroid medications with the use of beta-blockers to control symptoms (8). Methimazole and Propylthiouracil (PTU) are the two most common antithyroid medications used. There is, however, a significant incidence of allergic reactions to these medications. Urticaria and skin rashes have been reported to occur in 4-6% of patients within the first month of initiation of therapy (9). The skin reactions are typically mild and patients can potentially continue treatment with concurrent antihistamine therapy. In rare cases, patients may develop more severe reactions and need to discontinue medical management. In these circumstances, treatment with either surgery or radioactive iodine should be considered.

A number of cases have been reported in which acute thyrotoxic storm was successfully treated with thyroidectomy (10-13). In the majority of these cases, the patients were treated preoperatively with antithyroid medications. Hematologic complications secondary to PTU therapy have been reported which have required discontinuation of the medication prior to surgery (10). We report a case of a patient that developed angioedema of the face and resultant dyspnea. In a review of the literature, there were no additional cases reported in which patients in a thyroid storm underwent thyroidectomy due to methimazole-induced angioedema.

## 2. Case Presentation

A 30-year-old female admitted to an outside facility with urticaria, fevers, palpitations and tremors after recently being diagnosed with hyperthyroidism secondary to Graves’ disease. She had been having symptoms of anxiety, palpitations, weight loss and diarrhea for several months and was initiated on PTU and propranolol two weeks prior to admission. Her laboratory values on admission included a T3 resin uptake of 51%, T4 of 18.2 mcg/dL, free T4 of 5.46 ng/dL and a TSH of 0.01 U/mL. She presented an urticarial rash, palpitations and tremors, which were suggestive of thyroid storm with an associated allergic reaction to PTU. The PTU was discontinued, methimazole was initiated, and an increase in the dose of propranolol was made in an attempt to obtain symptomatic management. Despite the transition to methimazole, symptoms worsened and the patient developed angioedema of the face and progressive dyspnea. The methimazole was discontinued and the allergic reaction was addressed with dexamethasone, diphenhydramine and famotidine. The patient was then transferred to our institution for further management of her condition.

At the time of transfer, she was noted to have a diffuse urticarial rash over her face and extremities and was dyspneic at rest. A saturated solution of potassium iodide was started to help suppress further secretion of the thyroid hormone. The patient was not considered as a candidate for radioactive iodine because of concern for potential exacerbation of her condition and was subsequently scheduled for emergent thyroidectomy.

The patient had decadron and propranolol continued until the day of surgery. An esmolol infusion was used during surgery and titrated to the patient’s blood pressure. The patient received isoflurane, propofol, and a remifentanyl infusion during the procedure and remained hemodyanamically stable throughout the process. Total thyroidectomy was performed utilizing an endotracheal tube capable of nerve integrity monitoring to better enable preservation of the recurrent laryngeal nerves. Estimated blood loss was 50 ml. The parathyroid glands were adherent to the gland with three preserved and one requiring re-implantation. The patient was uneventfully extubated at the end of the case and recovered in the medical intensive care unit without complication. She experienced transient postoperative hypocalcemia (that resolved within two weeks after surgery). The patient was subsequently discharged on the second postoperative day with a dexamethasone taper, calcium, vitamin D and levothyroxine.

## 3. Discussion

Thyroid storm is a rare but dangerous condition that occurs in approximately 0.2% of all hyperthyroid patients (14). Rapid recognition and intervention is imperative. The use of antithyroid medications has been the mainstay of treatment. However, these medications also have the potential for significant side effects that must be anticipated and managed (15). Minor adverse reactions can occur in as many as 6% of patients, while more serious reactions are rare (9). In most cases, changing to another medical treatment option is sufficient yet this can be more challenging when the patient is in acute thyroid crisis.

Antithyroid medications may be used to convert patients to a euthyroid status before a procedure or provide definitive treatment for those who aren’t considered surgical candidates. Radioactive iodine is also an option in the treatment of hyperthyroidism, but is not a recommended treatment in the setting of acute thyroid storm because it can exacerbate the situation, take weeks to months to have full effect and has even induced thyroid storm in some cases (16). Thyroidectomy has been shown to be a reasonable treatment option for patients in thyroid crisis that can be treated preoperatively with antithyroid medications (10-13). In one case, a patient with Graves’ disease developed agranulocytosis from PTU requiring discontinuation of the medication, yet the patient was capable of being rendered euthyroid prior to surgery (10).

The successful surgical management of patients in thyroid storm, without obtaining euthyroid status prior to the procedure, has been reported (17). This series looked at 24 patients who had iodine-induced hyperthyroidism that had failed medical therapy and required surgical treatment. None of the patients in this series had hyperthyroidism prior to their iodine exposure nor did they have Graves’ disease. In this series, all of their patients but one survived. The series demonstrated, that although not without significant risk, hyperthyroid patients could undergo surgical thyroidectomy.

While the side effects of methimazole have been well described, there are only a limited number of reports describing treatment of patients in thyroid storm after an allergic reaction (9, 18). Fortunately, both thyroid storm in Graves’ disease and angioedema from methimazole are rare events. Medical management with antithyroid medications is the standard of care to re-establish a euthyroid state before more definitive treatment options are undertaken (8). Although this is the first line treatment, circumstances may arise that require alternative therapies. In such situations, emergent thyroidectomy has been shown to be a safe method for treating the crisis (8, 11-13).
